# The Corrosion Resistance of Reinforced Reactive Powder Concrete with Secondary Aluminum Ash Exposed to NaCl Action

**DOI:** 10.3390/ma16165615

**Published:** 2023-08-14

**Authors:** Hong Jiang, Kewei Wang, Hui Wang

**Affiliations:** 1 School of Municipal and Transportation Engineering, Anhui Water Conservancy Technical College, Hefei 231603, China; 2 School of Civil Engineering and Geographic Environment, Ningbo University, Ningbo 315000, China; wangkewei19@aliyun.com

**Keywords:** secondary aluminum ash, corrosion resistance, reinforced RPC, X-ray diffraction spectrum

## Abstract

Secondary aluminum ash (SAA) is a type of common solid waste which leads to pollution without treatment. Due to its chemical reactivity, the application of SAA to reactive powder concrete (RPC) may help solidify this solid waste while increasing its performance. However, RPC is usually in active service when used with steel bars. NaCl can corrode the steel bars when reinforced RPC is used in a coastal environment. In this study, the corrosion resistance of reinforced RPC was investigated. The specimens were exposed to an environment of NaCl with freeze–thaw cycles (F-Cs) and dry–wet alternations (D-As). The corresponding mass loss rates (MRs), the electrochemical impedance spectroscopy (EIS) curves and the dynamic modulus of elasticity (DME) were measured. The results show that the MR and the DME of reinforced RPC decrease with increasing values of F-C and D-A. F-C and D-A increases lead to increased electrical resistance (R). The real part value corresponding to the extreme point of the EIS curve is increased by 0~213.7% when the SAA is added. The relationship between the imaginary part and the real part of the EIS fits the quadratic function. The equivalent circuit of the reinforced RPC is obtained from the EIS curves. The R of the rust is calculated by using the equivalent circuit. The rust’s R decreases in the quadratic function with the mass ratio of the SAA. After 200 NaCl F-Cs, the MR, the DME and the R vary within the ranges of 23.4~113.6%, −2.93~−4.76% and 4.92~13.55%. When 20 NaCl D-As are finished, the MR, the DME and the R vary within the ranges of 34.7~202.8%, −13.21~−14.93% and 120.48~486.39%. The corrosion area rates are 2.3~68.7% and 28.7~125.6% higher after exposure to 200 NaCl F-Cs and 20 NaCl D-As. When the SAA is mixed, the MR is decreased by 0~13.12%, the DME increases by 0~3.11%, the R of the reinforced RPC increases by 26.01~152.43% and the corrosion area rates are decreased by 21.39~58.62%. This study will provide a novel method for solidifying SAA while improving the chlorine salt resistance of RPC.

## 1. Introduction

Secondary aluminum ash (SAA) is a solid waste which can induce relatively serious environmental pollution [1,2]. The main method used for the disposal of aluminum ash is the magnetic separation method, which can separate parts containing high-iron minerals from primary aluminum ash [3,4]. The disposed aluminum ash can be further recovered and utilized [5,6]. Moreover, plasma technology has been applied in treating aluminum ash. The magnetic separation method shows the advantage of extracting highly pure aluminum, but due to the need to wash the aluminum ash before magnetic separation treatment, a large amount of water will be consumed during the washing process, resulting in a significant waste of water resources and a long washing time, which is not conducive to industrialization [7,8]. The acid leaching method has been applied in the processing of SAA. Furthermore, fused salt electrolysis can be used for the disposal of SAA. However, the acid leaching method and fused salt electrolysis present longer times and higher costs [9,10]. Although many methods can be used to extract metal aluminum, the cost of processing is high, and many oxides have not been used in a timely manner. Hence, the resource utilization of SAA is very important.

SAA shows high chemical reactivity, which is advantageous for the performance of cement matrices [11,12]. Several studies have investigated the use of SAA in cement materials [13,14]. The reactive oxygen species can promote cement hydration, thereby enhancing the hydration degree of cement and the mechanical strength of cement concrete. Several studies on the influence of SAA on improving mechanical strength have been reported [15]. Although SAA is beneficial for concrete’s mechanical properties, the influence of SAA on the durability of cement concrete has attracted little attention from researchers.

Reactive powder concrete (RPC) is manufactured using a large number of mineral admixtures, quartz sand and other materials and is made according to the maximum density theory [16,17,18,19]. The influence of waste fly ash, furnace ash and rice husk ash on mechanical strength and resistance to chloride erosion have been investigated by prior researchers. The addition of waste fly ash, furnace ash and rice husk ash can increase flexural strength with maximum increasing rates of compressive strength of 36.1%, 27.4 and 31.2%, while the corresponding increasing rates are 23.4%, 18.6% and 24.3%. Meanwhile, the corresponding mass loss rates are 21.2%, 18.6% and 11.3% after 200 NaCl freeze–thaw cycles [20,21,22].

The application of SAA in RPC can help solidify this solid waste while increasing its performance [23]. However, RPC is usually used in actual engineering environments after the placement of steel bars. Steel bars are corroded by NaCl action when reinforced RPC is used in a coastal environment [24]. Previous studies have pointed out that solid waste possessing high active substances can improve the compactness of RPC, thus improving the corresponding mechanical strength and the corrosion resistance [25,26]. When reinforced RPC is used in marine environments, the steel bars’ inner RPC is easily corroded by chloride ions in seawater [27]. The addition of SAA may have complex impacts on the corrosion resistance of reinforced RPC. However, little attention has been paid to this research direction.

In this study, the effect of SAA on the corrosion resistance of reinforced RPC is investigated. The specimens are exposed to a NaCl corrosion environment. The mass loss rates (MRs), AC electrical resistance (R), electrochemical impedance spectroscopy (EIS) curves and dynamic modulus of elasticity (DME) of reinforced RPC are determined for the characterization of corrosion resistance. This study will provide a reference for the use of reinforced RPC with secondary aluminum ash in marine environments.

## 2. Materials and Methods

### 2.1. Raw Materials

For clarity, the abbreviations of all nouns in the text are presented in Table 1. Ordinary Portland cement (OPC) provided by Guizhou Kunhao Yongsheng Trading Co., Ltd., Guiyang, China, was used for manufacturing the RPC. The OPC showed initial and final setting times of 121.4 min and 354.3 min.

Fly ash (FA) with a purity higher than 99% was provided by Emeishan Taixin Environmental Protection Technology Co., Ltd., Leshan City, China. The FA had high corrosion resistance, showing a density of 1.5 g/cm^3^ and a particle size of 0.05–0.20 μm. Secondary aluminum ash (SAA) with Al_2_O_3_ content ranging from 20% to 60% and SiO_2_ content below 8% was purchased from Anyang County Jiacheng Yenai Co., Ltd., Anyang, China. Quartz sand produced by Changxing Qingsheng Calcium Industry Co., Ltd., Changzhou, showed a SiO_2_ content of 99.7% and apparent density of 2.66 g/cm^3^. Level S95 blast furnace slag powder (BFS) with a density of 2.88 g/cm^3^, activity index above 95%, a specific surface area of 437.1 m^2^/g and a loss on ignition of 2.21%, manufactured by Hebei Chuangtian Engineering Materials Co., Ltd., Shijiazhuang, China, was used as another mineral admixture. Three scales of quartz sand with particle sizes of 3.96~1.39 mm, 0.83~0.42 mm and 0.35~0.17 mm and with mass ratios of 1:1.5:1 were used as the aggregate. Liquid polycarboxylate water reducer with a solid content of 23.6% was provided by Anhui Huashi Nanotechnology Co., Ltd., Hefei, China, showing a water-reducing rate of 40.1%. The particle size and compositions of the cementitious materials are shown in Table 1 and Table 2. The flowability of fresh RPC was adjusted by polycarboxylate superplasticizer, whose water-reducing rate was 37.8%.

### 2.2. The Manufacturing Process of Specimens

**Figure 1:**
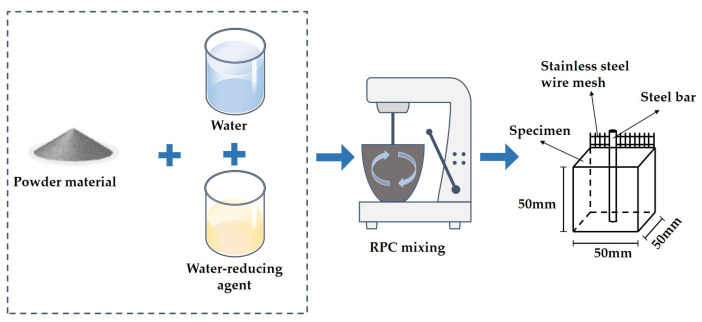
The preparation of RPC specimens.

A UJZ-15 mixer was used for stirring the RPC. The dry materials were added to the mixer and stirred with a mixing speed of 60 ± 2 r/min for 2 min, and then water mixed with a water-reducing agent was added to the mixer. A mixing speed of 80 ± 2 r/min was used for manufacturing the RPC. Specimens with a size of 50 × 50 × 50 mm^3^ were used for the measurement of corrosion resistance. Steel bars and stainless steel wire mesh were used as the two electrodes of the specimen. The stainless steel wire mesh was vertically inserted into the back of the specimen and 5 mm away from the edge. The steel bars were vertically inserted into the center of the specimen, and the center of the steel bars was 25 mm away from the boundary of the specimen. The specimens were cured in the standard curing environment of 20.3 °C and relative humidity of 96.2%. Figure 1 shows the manufacturing process of the RPC samples. Meanwhile, the mixing proportions of SAA-RPC are shown in Table 3. Six specimens were used in each experiment.

### 2.3. NaCl Action on RPC

The specimens were immersed in a NaCl solution with 3% mass fraction for 4 days after 24 days’ standard curing. After this, the specimens were moved to the rapid freeze–thaw machine provided by Tianjin Gangyuan Test Instrument Factory, Tianjin, China, for the rapid freeze–thaw (F-C) test. A temperature range of −15 °C~ 8 °C was used for the experiment. The specimens were placed in plastic sleeves, which were filled with 3% NaCl solution. During the F-C test, the specimens underwent a total of 200 cycles, and specimens were taken out every 50 cycles to measure the MR, DME and R. Each F-C lasted for 3 h (2 h freezing and 1 h thawing). A dry–wet cyclic salt spray tester from Jiangsu Jiuyi Power Equipment Co., Ltd., Yangzhou, China, was used in the measurement of NaCl dry–wet (D-A) alternations. The MR, DME and R of specimens were measured after every 5 alternations. Each D-A cycle consisted of three steps totaling 48 h. The specimens with no excess water on the surface were dried at 80 °C for 36 h, and then the dried specimens were cooled for 2 h. Finally, the cooled specimens were immersed in NaCl solution for 10 h. After the measurements were taken, all specimens were returned. The measuring process lasted for a very short time, and the conditions for the specimens were not changed. Similar experimental methods can be found in the Chinese standard GB/T50082-2009 and Cao et al.’s studies [28,29].

### 2.4. The Corrosion Resistance Parameters

The initial masses of specimens were weighed using an electronic scale after being immersed in the NaCl solution for 4 days. The specimens were cleaned with water and wiped with a cloth after the NaCl D-A cycles. After NaCl exposure, the mass was weighed, and the mass loss rate (*MR*) was calculated using Equation (1) [29], where *m*_1_ is the initial mass of the RPC before NaCl action and *m*_t_ is the mass of the RPC after a certain number of NaCl F-Cs or D-As.

(1) $$MR=\frac{m_{1}-m_{t}}{m_{1}}$$

An HC-U81 concrete ultrasonic detector (provided by Zhongjiao Jianyi Precision Instrument Co., Ltd., Beijing, China) was used for testing the dynamic modulus of elasticity (*DME*). The definition of dynamic moduli of elasticity can be found in the References and can be obtained from Equation (2) [30], where *V*_1_ is the initial ultrasonic velocity before NaCl action and *V*_t_ is the ultrasonic velocity of specimens after a certain number of NaCl F-Cs or D-As.

(2) $$DME={\left(\frac{V_{t}}{V_{1}}\right)}^{2}\times 100\%$$

**Figure 2:**
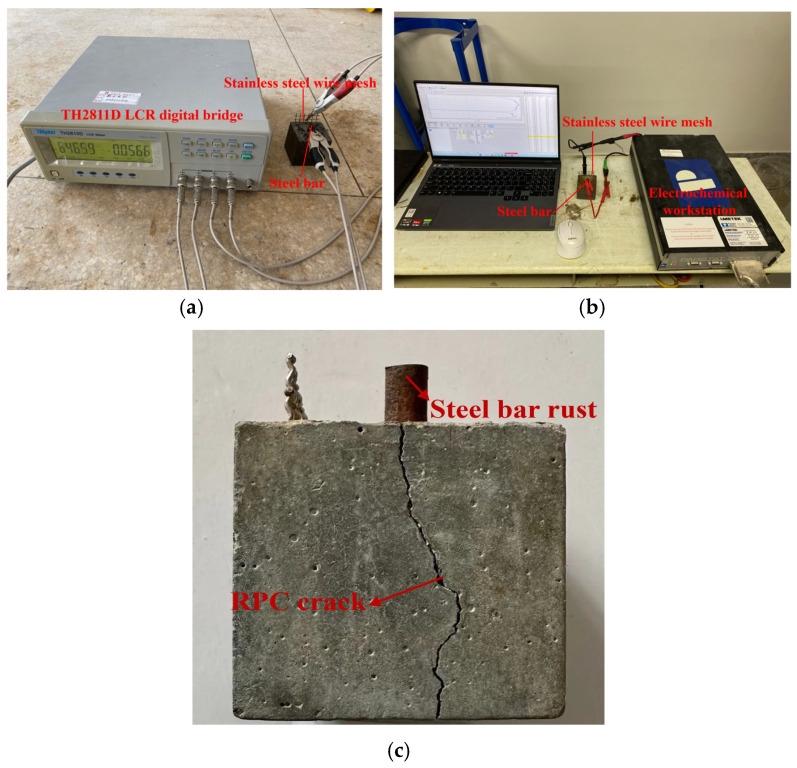
The measuring process of electrical parameters. (**a**) The measurement of R; (**b**) the measurement of EIS and Tafer curves; (**c**) the specimens after NaCl action.

A TH2811D LCR digital bridge was used for the measurement of the R of RPC with a testing frequency of 10^4^ Hz and a voltage of 1 V. A Zhuodi electrochemical workstation substance qualitative and quantitative analysis instrument, purchased from Zhuodi Instrument Equipment Co., Ltd., Shanghai, China, was used for the measurement of EIS with an AC voltage ranging from −10 mV to 10 mV. Steel bars and stainless steel wire mesh were used as the two electrodes of the specimen in the experiment. The voltage applied to the Tafer curves ranged from −0.15 V to 0.15 V. Due to the high resistance of the sample, the current magnitude inside the sample during testing was only within the range of 10^−4^~10^−6^ A/cm^2^, and the dynamic potential scanning process lasted for 5.96 s. The additional corrosion caused to the sample can be almost negligible, so it can be considered as non-destructive testing. The equipment measuring processes of the R and the EIS curves are shown in Figure 2. In this study, six specimens were selected for the corrosion resistance experiment. The average value of six specimens was considered to be the experimental result. Table 4 shows the number of specimens and the standard deviation for testing parameters. The measurement details can be found in Cao’s study [31].

## 3. Results and Discussion

When the steel bars of RPC corrode, the rust will increase the cracking of RPC, leading to an increase in the spalling of RPC. The increased cracks will prevent the propagation speed of electrons and sound waves, which will result in a decrease in the relative dynamic elastic modulus and an increase in electrical resistance. Therefore, the mass loss rate, the dynamic modulus of elasticity and the electrical resistance of reinforced RPC were measured to reflect the corrosion resistance of reinforced RPC.

### 3.1. The Mass Loss Rate

**Figure 3:**
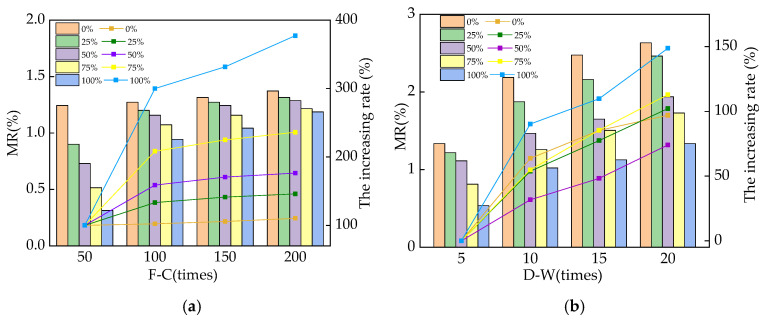
The MR after different numbers of NaCl F-Cs and D-As. (**a**) After exposure to different F-Cs; (**b**) after exposure to different D-As.

The mass loss rates (MRs) of the reinforced RPC after different cycles of NaCl F-C and D-A are illustrated in Figure 3. In Figure 3, the left y-axis represents the mass loss rate, while the right y-axis represents the growth rate of the mass loss rate with the increasing cycles. The MR increases with an increasing number of NaCl F-Cs and D-As. This is ascribed to the fact that the NaCl F-C and D-A action can increase the crystallization stress of RPC by more than 200% and 300%, respectively, leading to an increase in spalling on the surface of the RPC specimens. Moreover, as the NaCl D-A cycle continues, the number of NaCl crystals in the RPC increases, which leads to the growth of the crystalline stress in the RPC and leads to the loss of the RPC specimen’s mass. In addition, the NaCl D-A cycles will lead to the enrichment of the NaCl solution in the pore solution of the RPC. After the specimen is dried, the moisture evaporates, and the crystallization of NaCl in RPC induces crystallization stress, which can cause RPC to crack and peel off, leading to a decrease in RPC mass. Furthermore, as shown in Figure 3, the addition of SAA can decrease the MR of the RPC after NaCl action. This is ascribed to the pozzolanic effect by the SAA in RPC, which can decrease the mass loss rate (MR) of RPC after NaCl F-Cs and D-As [32,33]. After 200 NaCl F-Cs and 20 NaCl D-As, the MR of RPC is decreased by 23.4~113.6% and 34.7~202.8%, respectively.

### 3.2. The Dynamic Modulus of Elasticity of Reinforced RPC

**Figure 4:**
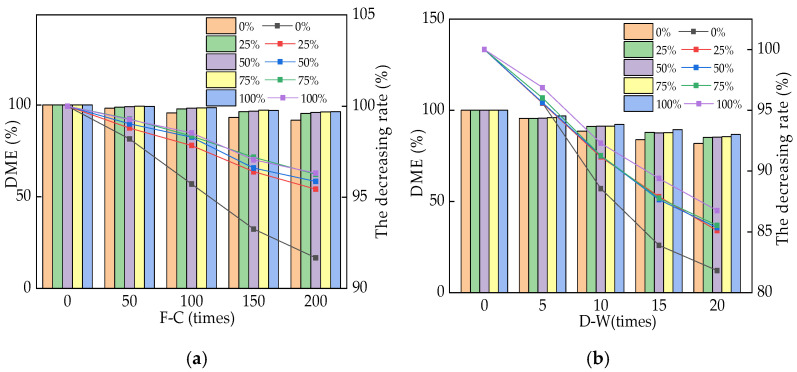
The DME of RPC exposed to NaCl action. (**a**) The DME of RPC exposed to different F-Cs; (**b**) the DME of RPC exposed to different D-As.

Equation (2) was applied to calculate the dynamic modulus of elasticity (DME) of the reinforced RPC in this study. The DME of reinforced RPC during NaCl F-Cs and D-As is shown in Figure 4. In Figure 4, the left y-axis represents the value of the dynamic modulus of elasticity (DME), while the right y-axis represents the loss rate of DME with the increasing cycles. The DME of RPC decreases with the increasing number of NaCl F-Cs and D-As. The frost heave stress and pore pressure caused by the NaCl F-Cs and the crystallization stress caused by the NaCl D-As can accelerate the number and expansion of cracks inside RPC, which can block the propagation of ultrasound [34,35]. Therefore, the DME decreased with an increasing number of NaCl F-Cs and D-As. After 200 NaCl F-Cs and 20 NaCl D-As, the DME was increased by adding SAA. As described in Wang’s studies, NaCl F-Cs and D-As can accelerate the number and width of cracks, which prevents the transmission of ultrasound and decreases the DME. Moreover, the chloride ions will penetrate into the RPC along the cracks to corrode the steel bars, further accelerating the expansion of the cracks and reducing the DME value. The DME is decreased with the decreasing rates of 2.93~4.76% and 13.21~14.93% after the effect of 200 NaCl F-Cs and D-As. Compared with the RPC mixed with waste fly ash, the DME of SAA-RPC was 1.3~2.6% higher after 200 NaCl F-Cs and 2.1~3.3% higher after 20 NaCl D-As [36,37,38].

### 3.3. The Electrical Parameters

**Figure 5:**
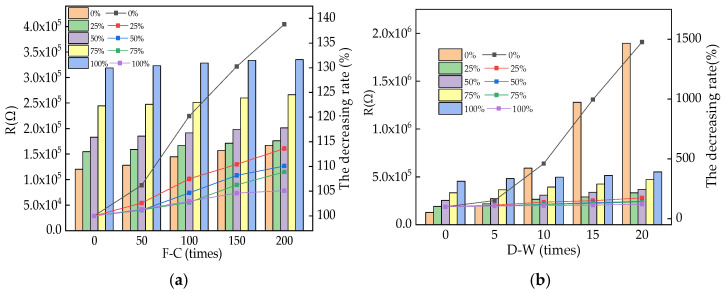
The R of specimens after various numbers of NaCl effects. (**a**) After exposure to different F-Cs; (**b**) after exposure to different D-As.

The electrical resistance (R) of RPC during the NaCl F-Cs and D-As is shown in Figure 5. In Figure 5, the left y-axis represents the value of R, and the right y-axis represents the loss rate of R with the cycle increases. As shown in Figure 5, the R increases with the increasing numbers of NaCl F-Cs and D-As. This is ascribed to the fact that the NaCl F-Cs and D-As can accelerate the increase and expansion of internal cracks in RPC [39]. The increased inner cracks delay the propagation of electrons in the RPC. Therefore, the R is decreased by NaCl action, leading to an increase in the R of reinforced RPC [40,41]. When the dosages of SAA are increased, the initial R is increased and the increasing rates of R are decreased, indicating that the corrosion resistance of reinforced RPC is improved by adding the SAA. Moreover, the RPC specimens subjected to the NaCl D-A effect showed a higher R rate than the RPC specimens subjected to the NaCl F-C effect, indicating that when the NaCl D-A effect is exerted on the reinforced RPC, the reinforcement corrodes more seriously than in the specimens subjected to NaCl F-Cs. After 200 NaCl F-Cs or 20 NaCl D-As, the AC electrical resistance increased by the rates of 4.92~13.55% and 120.48~486.39%, respectively. The AC electrical resistance was increased by 26.01~152.43% after the SAA was added.

**Figure 6:**
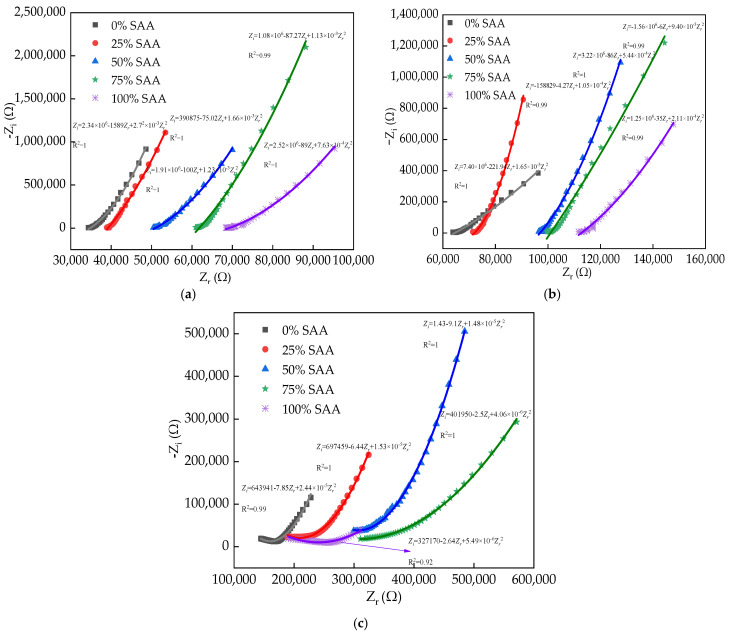
The EIS curves of reinforced RPC. (**a**) Before NaCl erosion; (**b**) after NaCl F-C; (**c**) after NaCl D-A.

Figure 6 shows the EIS curves of the reinforced RPC. In Figure 6, *Z_i_* represents the electrical reactance of reinforced RPC and *Z_r_* represents the corresponding electrical reactance. The relationship between *Z_i_* and *Z_r_* accords with quadratic function. The real part values corresponding to the extreme point of the EIS curves increase with the NaCl D-A and NaCl F-C effects. Moreover, the increasing amounts of SAA lead to the minimum points being increased. This is attributed to the increased F-C or D-A damage caused by NaCl D-A or NaCl F-C action [42]. Consequently, the R is increased. The R-squared values of the reinforced RPC’s EIS curves are 0.99 and 1.0, showing the reasonability of the fitting results.

**Figure 7:**
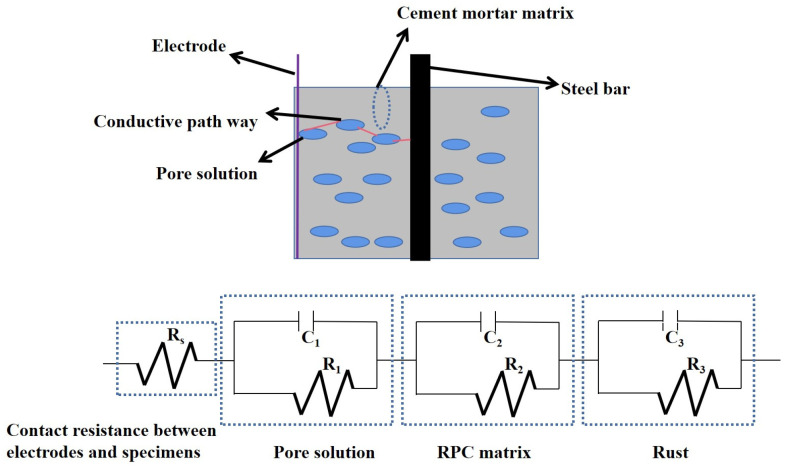
The equivalent circuit of reinforced RPC.

The equivalent circuit of reinforced RPC is shown in Figure 7. It can be seen in Figure 7 that the equivalent circuit of reinforced RPC consists of four electrical components. *R*_0_ in the equivalent circuit represents the contact electrical resistance between the RPC matrix and the steel bar or the electrode, where *R_i_* and *C_i_* (*i* = 1, 2, 3) are the parallel resistances and capacitances of the pore solution, the rust on the surface of the steel bars and the RPC matrix. In Figure 7, *R*_0_ and the parallel resistances and capacitances of the pore solution, the rust on the surface of the steel bars and the RPC matrix are in series. The specific analysis of equivalent circuit diagrams can be found in the studies of Wang et al. [43].

**Figure 8:**
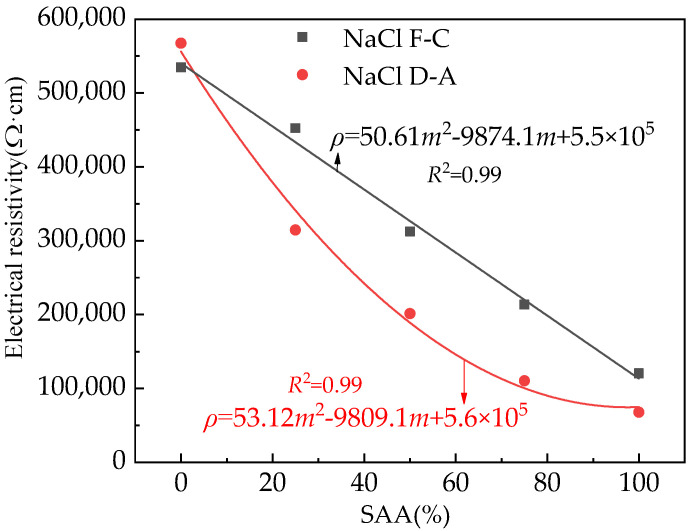
The R of rust of the reinforced RPC.

The electrical resistivity of the rust is shown in Figure 8. In Figure 8, *m* means the ratio of SAA mass to the total mass of SAA and FA. As illustrated in Figure 8, the electrical resistivity of the rust increases with the increasing number of NaCl F-Cs and D-As. This is ascribed to the fact that the NaCl action can accelerate the steel bars’ corrosion, increasing the rust’s electrical resistivity [44]. Therefore, the NaCl action can accelerate the steel bars’ corrosion. Moreover, the increasing content of SAA can decrease the electrical resistivity of the rust, showing the fact that the SAA can improve the corrosion resistance of the reinforced RPC. Moreover, as shown in Figure 8, the rust’s electrical resistivity of the reinforced RPC after exposure to NaCl D-A action is higher than the rust’s electrical resistivity of the reinforced RPC after the NaCl F-C effect. The R-squared of the relationship between the electrical resistivity and the mass ratio of SAA is 0.99, which shows the accuracy of the fitting equations.

**Figure 9:**
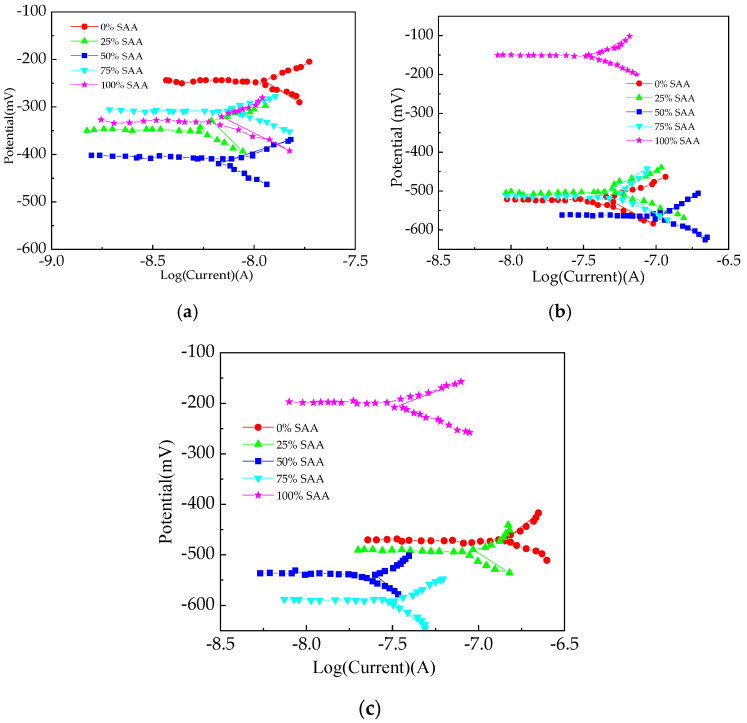
The Tafel curves of reinforced RPC. (**a**) The Tafel curves of reinforced RPC before NaCl action; (**b**) the Tafel curves of reinforced RPC after NaCl F-C; (**c**) the Tafel curves of reinforced RPC after NaCl D-A.

The Tafel curves of reinforced RPC under the NaCl F-Cs and D-As are shown in Figure 9. In Figure 9, the percentage means the proportion of the SAA mass to the total mass of SAA and FA. The voltage range applied to the electrode was −0.15 V to 0.15 V. Due to the high resistance of the specimen, the current inside the specimen during testing was very small, and the linear polarization scanning process took an extremely short time. The Tafel curves can be divided into two parts. Firstly, the electrical voltage of reinforced RPC is maintained at horizontal relationships with the logarithm of current. Then, the Tafel curves are changed into piecewise functions. As shown in Figure 9, the extreme points of the potential voltage range from −623 mV to −200 mV, and the addition of SAA can decrease the potential voltage values corresponding to the extreme points (each Tafel curve is composed of two curves; the bifurcation point of the two curves is the extreme point). Due to the fact that the addition of SAA can form calcium aluminate, which increases the compactness of the RPC hydration products, the penetration of chloride ions is prevented, leading to the corrosion resistance of reinforced RPC being improved. Moreover, as shown in Figure 9, the reinforced RPC exposed to NaCl D-A action shows higher extreme point values of the potential voltage than that exposed to NaCl F-Cs.

The corrosion area rate of steel bars can be obtained by the Tafel curves as expressed in Equation (3) [45].

(3) $$v=\frac{10m}{\frac{9.65\times 10^{4}}{3.6}N}i=3.73\times 10^{-4}\frac{m}{N}i$$

where *v* is the corrosion rate of inner steel bars with the unit of g/m^2^h, *m* means the atomic weight of the metal with the unit of g, *N* represents the atomic valence of the metal and *i* represents the corrosion current density, whose unit is μA/cm^2^.

**Figure 10:**
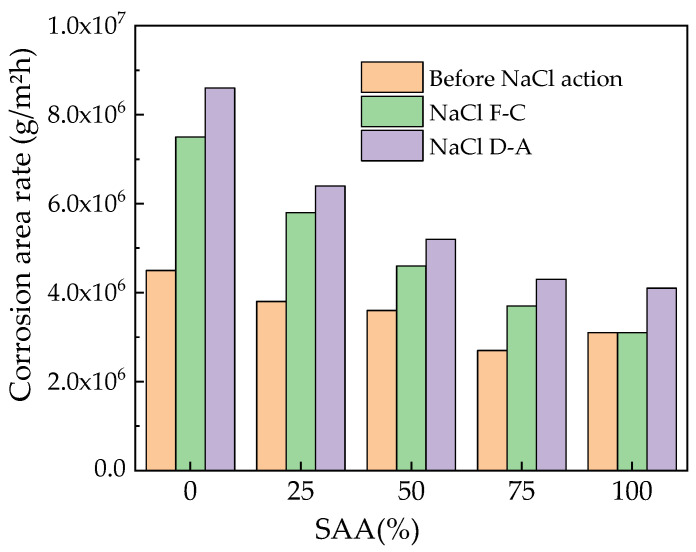
The corrosion rate of the steel bars.

The corrosion area rate of the reinforced RPC’s inner steel bars is shown in Figure 10. As depicted in Figure 10, the corrosion area rate of the reinforced RPC’s inner steel bars increases with the increase in NaCl F-Cs and D-As. Moreover, the addition of SAA demonstrates a decreasing effect on the corrosion area rate of the reinforced RPC’s inner steel bars, thus indicating the fact that SAA can improve the corrosion resistance of reinforced RPC. Furthermore, as illustrated in Figure 10, the corrosion area rate of the reinforced RPC’s inner steel bars exposed to NaCl D-A is higher than that of specimens exposed to NaCl F-C.

## 4. Conclusions

The corrosion resistance of reinforced RPC with SAA was investigated in this study. The NaCl F-Cs and D-As were considered.

The mass of the reinforced RPC decreased during the NaCl F-C and D-A action. It was shown that 200 NaCl F-Cs and 20 NaCl D-As can increase the MR by 23.4~113.6% and 34.7~202.8%, respectively. Moreover, the DME was decreased by rates of 2.93~4.76% and 13.21~14.93%. The addition of SAA helped decrease the MR and increase the DME of the reinforced RPC. The MR was decreased by 0~13.12% and the DME was increased by 0~3.11% when SAA was added.

The AC electrical resistance of reinforced RPC before NaCl action was increased by adding the SAA. The NaCl F-Cs and D-As led to an increase in the electrical resistance of the reinforced RPC. After 200 NaCl F-Cs or 20 NaCl D-As, the AC electrical resistance increased by rates of 4.92~13.55% and 120.48~486.39%, respectively. The AC electrical resistance was increased by 26.01~152.43% after the SAA was added.

The AC electrical reactance varied in the quadratic function with the AC electrical resistance. The equivalent circuit of the reinforced RPC consisted of the contact electrical resistance between the electrode and the RPC matrix in series with three electrical components. The three electrical components were the parallel electrical resistance and reactance of the pore solution, the RPC matrix and the rust. The electrical resistance of the rust on the surface of steel bars was increased after exposure to NaCl action and decreased by adding the SAA.

The Tafel curves’ results show that the SAA can decrease the corrosion current, thus improving the corresponding corrosion resistance. The reinforced RPC corroded more seriously with the NaCl action. The NaCl D-As generated more severe corrosion on the reinforced RPC than the NaCl F-Cs. The addition of SAA decreased the corrosion area rates of the steel bars reinforced by RPC by 21.39~58.62%.

The coupling effect of multiple environments on the corrosion resistance of the reinforced SAA-RPC was not considered and will be studied in the future.

## Figures and Tables

**Table 1:** The accumulated pass rate of the powder materials (%).

Types	Particle Size/μm						
	0.3	0.6	1	4	8	64	360
OPC	0.13	0.33	2.36	15.22	28.25	93.11	100.00
BFS	0.04	0.12	3.25	19.31	35.08	97.86	100.00
FA	31.14	58.33	82.17	100.00	100.00	100.00	100.00
Quartz sand	0.00	0.00	0.00	0.00	0.04	23.93	100.00
SAA	0.06	0.24	0.56	1.15	3.92	25.9	87.25

**Table 2:** Composition of the powder materials (%).

Types	SiO_2_	Al_2_O_3_	Fe_x_O_y_	MgO	CaO	SO_3_	K_2_O	Na_2_O	Ti_2_O	Loss on Ignition
OPC	20.77	5.47	3.88	1.69	61.83	2.68	-	-	-	3.08
BFS	33.8	15.1	0.6	9.7	36.7	0.4	3.7	-	-	-
FA	90.60	0.23	0.61	0.25	0.43	0.20	7.50	-	-	-
Quartz sand	99.9	-	0.1	-	-	-	-	-	-	-
SAA	4.6	79.3	3.9	5.7	1.5	-	-	0.9	-	-

**Table 3:** The mixing proportions of RPC (kg/m^3^).

Water	OPC	SAA	FA	BFS	Quartz Sand	Water Reducer
241.96	733.29	0.00	366.60	109.99	968.12	16.14
241.96	733.29	91.67	274.92	109.99	968.12	16.14
241.96	733.29	183.35	183.35	109.99	968.12	16.14
241.96	733.29	274.92	91.67	109.99	968.12	16.14
241.96	733.29	366.60	0.00	109.99	968.12	16.14

**Table 4:** The number of specimens and the standard deviation for testing parameters.

Measuring Parameters	Number of Specimens	Standard Deviation
MR	6	0.091
DME	6	0.087
R	6	0.093
EIS	6	0.098
Tafel experiment	6	0.093

## Data Availability

Not applicable.

## Abbreviations

OPC	Ordinary Portland cement
F-C	Freeze–thaw cycle
D-A	Dry–wet alternation
MR	Mass loss rate
EIS	Electrochemical impedance spectroscopy
DME	Dynamic modulus of elasticity
R	AC electrical resistance
SAA	Secondary aluminum ash
BFS	Blast furnace slag
FA	Fly ash
